# Developing Clinical Strength-of-Evidence Approach to Define HIV-Associated Malignancies for Cancer Registration in Kenya

**DOI:** 10.1371/journal.pone.0085881

**Published:** 2014-01-23

**Authors:** Anne Korir, Nathan Mauti, Pamela Moats, Matthew J. Gurka, Geoffrey Mutuma, Christine Metheny, Peter M. Mwamba, Peter O. Oyiro, Melanie Fisher, Leona W. Ayers, Rosemary Rochford, Walter O. Mwanda, Scot C. Remick

**Affiliations:** 1 Kenya Medical Research Institute, Nairobi Cancer Registry, Nairobi, Kenya; 2 West Virginia University Hospitals, Health Information Management–Cancer Registry, Morgantown, West Virginia, United States of America; 3 Department of Biostatistics, West Virginia University School of Public Health, Morgantown, West Virginia, United States of America; 4 University of Nairobi Institute of Tropical and Infectious Diseases (UNITID), University of Nairobi College of Health Sciences, and Kenyatta National Hospital, Nairobi, Kenya; 5 Department of Medicine, Global Health Program (MF) and Mary Babb Randolph Cancer Center (SCR), West Virginia University School of Medicine, Morgantown, West Virginia, United States of America; 6 Department of Pathology, The Ohio State University College of Medicine, Columbus, Ohio, United States of America; 7 Department of Microbiology and Immunology, SUNY Upstate Medical University, Syracuse, New York, United States of America; University of Southern California Keck School of Medicine, United States of America

## Abstract

**Background:**

Sub-Saharan Africa cancer registries are beset by an increasing cancer burden further exacerbated by the AIDS epidemic where there are limited capabilities for cancer-AIDS match co-registration. We undertook a pilot study based on a *“strength-of-evidence”* approach using clinical data that is abstracted at the time of cancer registration for purposes of linking cancer diagnosis to AIDS diagnosis.

**Methods/Findings:**

The standard Nairobi Cancer Registry form was modified for registrars to abstract the following clinical data from medical records regarding HIV infection/AIDS in a hierarchal approach at time of cancer registration from highest-to-lowest strength-of-evidence: 1) documentation of positive HIV serology; 2) antiretroviral drug prescription; 3) CD4+ lymphocyte count; and 4) WHO HIV clinical stage or immune suppression syndrome (ISS), which is Kenyan terminology for AIDS. Between August 1 and October 31, 2011 a total of 1,200 cancer cases were registered. Of these, 171 cases (14.3%) met clinical strength-of-evidence criteria for association with HIV infection/AIDS; 69% (118 cases were tumor types with known HIV association – Kaposi’s sarcoma, cervical cancer, non-Hodgkin’s and Hodgkin’s lymphoma, and conjunctiva carcinoma) and 31% (53) were consistent with non-AIDS defining cancers. Verifiable positive HIV serology was identified in 47 (27%) cases for an absolute seroprevalence rate of 4% among the cancer registered cases with an upper boundary of 14% among those meeting at least one of strength-of-evidence criteria.

**Conclusions/Significance:**

This pilot demonstration of a hierarchal, clinical strength-of-evidence approach for cancer-AIDS registration in Kenya establishes feasibility, is readily adaptable, pragmatic, and does not require additional resources for critically under staffed cancer registries. Cancer is an emerging public health challenge, and African nations need to develop well designed population-based studies in order to better define the impact and spectrum of malignant disease in the backdrop of HIV infection.

## Introduction

The global burden of cancer is assuming the mantle as a worldwide public health problem. Estimates are that by 2010 cancer became the world’s single leading cause of death. [1]–[4] This is substantiated by an estimated 12.7 million new cases and 7.6 million deaths based on Globocan 2008, which contributed 13% of the global mortality burden. [1], [2], [5]–[7] This eclipsed the total number of deaths attributable to HIV infection/AIDS, tuberculosis and malaria combined. [8] The World Health Organization (WHO) projects that by 2020 there will be 16 million and by 2030, 27 million new cancer cases, 70% of these will be in developing nations, and an excess of 1 million cases will occur in sub-Saharan Africa. [7], [9]–[12] The cancer burden in the sub-Saharan Africa region will continue to be complicated by the AIDS epidemic.

As of December 2009, it was estimated that 33.3 million people were living in the world with HIV infection, there are 2.6 million newly infected persons, and there are 1.8 million deaths that are attributable to HIV/AIDS. [13], [14] More than 95% of those living with HIV disease reside in developing countries, and of these two-thirds (22.5 million) reside in sub-Saharan Africa. [13]–[15] In sub-Saharan Africa the adult HIV seroprevalence rate is on the order of 5.2% [15].

In Kenya, a country with a population of nearly 41 million people, there are limited published data on cancer burden and essentially no data on the impact of HIV infection on the incidence of AIDS-related neoplasms. [16] Nairobi is the 121^st^ largest city in the world; the 8^th^ largest in sub-Saharan Africa; and Kenya has the 3^rd^ highest population of persons living with HIV infection or AIDS (PLWA) in Africa trailing only South Africa and Nigeria. [15]–[17] The Nairobi Cancer Registry (NCR), established in 2001, is the longest serving population-based registry (∼3 million persons) in Kenya; is housed in the Pathology and Oncology Research Unit, Centre for Clinical Research at the Kenya Medical Research Institute (KEMRI) in Nairobi; and is a flagship activity of the Non-Communicable Diseases Research Programme. The registry captures tumor registration from 24 healthcare entities [e.g., Kenyatta National Hospital (KNH) the essential national referral center in Nairobi; mission hospitals, private hospitals, and hospice; other healthcare organizations and clinics, private physician offices, and other entities all of which are located in Nairobi, Kenya.][18] Not unlike other tumor registries in sub-Saharan Africa reporting periods and tumor registration are often interrupted due to a variety of reasons; and many nations either have nascent tumor registries or no registries at all. [18] In Kenya, the HIV seroprevalence rate has fallen from 14% in the mid-1990s to 6.3% in 2009. [16] In 2009, it was estimated that 1.5 million Kenyans were living with HIV/AIDS inclusive of 6.3% adults aged 15 and up; there were 180,000 children (aged 0–14 years) living with HIV; and there were 80,000 deaths estimated due to AIDS. [16] The NCR has been unable to link HIV incidence data, although HIV is a reportable disease in Kenya, to cancer registration; there is no computerized capability for registration; and the registry must expand regionally and nationally to provide more meaningful data. Similar challenges with cancer and HIV infection co-registration or matching are encountered in other sub-Saharan African nations as well. We undertook a pilot study based on a *“strength-of-evidence”* approach using clinical data that is abstracted at the time of cancer registration for purposes of linking cancer diagnosis to HIV infection/AIDS diagnosis.

## Case Ascertainment and Methods

### Cancer Registration

The NCR is a population registry and uses the National Bureau of Statistics to define the census population as the denominator for calculating incidence rates. Anyone who has lived/worked in Nairobi in the past 6 months is considered a resident. For this preliminary study, cancer registration (there was no specified time period for cancer diagnosis) was undertaken in a manner consistent with KEMRI/NCR operating policies and procedures. This includes scientific and ethical approval from the KEMRI Scientific Steering Committee and Ethical Review Committee in addition a letter from the Ministry of Health authorizing access to medical records is also obtained. Given these procedures, informed consent for tumor registration and data abstraction is not obtained. All data was de-identified prior to analyses.

### HIV/AIDS Association and Case Ascertainment

The standard NCR cancer registration form (CanReg4 that is being updated with CanReg 5) [19], [20] was modified for tumor registrars to abstract the following clinical data regarding HIV infection/AIDS in a hierarchal approach from the medical records at time of cancer registration from highest-to-lowest strength-of-evidence: 1) documentation of positive or negative HIV serology test [i.e., commonly employed rapid HIV serology tests in Kenya include: HIV-1/2 3.0 Rapid Test SD, Bioline (London, UK); Uni-Gold™ Recombigen®, Trinity Biotech PLC (Wicklow, Ireland); and Determine® HIV-1/2 Rapid Test, Abbott Laboratories (Abbott Park, IL, USA)]; 2) documentation of combination antiretroviral therapy (cARV) and/or the precise regimen in the medical record; 3) laboratory report of CD4+ lymphocyte counts and, 4) documentation in the medical record of WHO clinical staging (I through IV) of HIV disease and/or medical record documentation of immune suppression syndrome also commonly referred to as immunosuppression syndrome or ISS, which is the acronym for AIDS in Kenya. The modified NCR form underwent ethical committee review at the KEMRI for the pilot data reporting period, and was approved for use. All aspects of cancer disease registration and capturing of HIV/AIDS clinical data strictly adhered to Kenyan national ethical guidelines. A second attempt to capture additional HIV serology data was undertaken after initial data review in October 2011. In this instance, NCR registrars were referred to the Comprehensive Care Clinic, a multidisciplinary treatment center for management of patients with HIV infection/AIDS and the Department of Radiation Oncology both located on the KNH campus.

### Statistical Considerations

Comparisons were made between those individuals classified solely by clinical criteria and those who had positive HIV serology (documented on a HIV serology report contained in the medical record) using a number of characteristics, including strength-of-evidence criteria, patient demographics, and proportion with tumors known to be associated with HIV infection. Rates between the two groups were compared via Chi-square tests, while age was compared with a t-test (α = 0.05).

## Results

### Case Ascertainment

From August 1 to October 31, 2011 a total of 1,200 cancer cases were registered from 10 centers, and subjected to verification for HIV/AIDS association. Of these, 171 cancer cases (14.3%) met clinical strength-of-evidence criteria for association with HIV infection or AIDS (Table 1). These cases were diagnosed between January 2006 and July 2011; 123 cases (72%) were diagnosed in 2010 and 2011.

**Table 1:** Nairobi Cancer Registry (NCR) – reporting entities comprising this cancer population-based registry utilized for this study number of cases that met strength-of-evidence criteria for cancer-AIDS (HIV infection) match.

Reporting Healthcare Entityin Nairobi	Nos. HIV CasesIdentified (%)
Aga Khan University Hospital	1 (<1%)
Avenue Hospital	3 (2%)
Cancer Care Kenya	5 (3%)
Private physician clinic(s)	1 (<1%)
Karen Hospital	6 (4%)
Kenyatta National Hospital	86 (50%)
Metropolitan Hospital	2 (1%)
St. Mary’s Hospital	25 (15%)
Nairobi Hospice	31 (18%)
Nairobi West Hospital	2 (1%)
Registry of Births and Deaths, Nairobi	9 (5%)
Total	171 (100%)

### Strength-of-evidence of HIV/AIDS Association

Working from the hierarchal approach defined above, 25 (14.6%) of the 171 cases had definitive documentation of HIV infection with laboratory reports confirming positive HIV serology. Of the remaining 146 clinical cases, 74 (50.7%) had documentation of combination antiretroviral therapy; 28 (19.2%) had documentation of CD4+ lymphocytes counts ≤350 cells/µL [any CD4+ count was documented in 41 (28.1%) with a range 21–1007 cells/µL]; and all 146 cases without documentation of positive HIV serology fulfilled some of criteria listed above and/or documentation of immunosuppression syndrome including WHO clinical stage in the medical record. Table 2 summarizes the composite data of the strength-of-evidence criteria utilized on the basis of positive HIV serology vs. other clinical criteria; it is noted these criteria are not mutually exclusive.

**Table 2:** Level of strength-of-evidence of HIV infection/AIDS in 171 cancer cases that were registered.

		HIV Serology			
Level Strength-of-Evidence	Total(n = 171)	Initial HIV(+) Serology (n = 25)	Subsequent ConfirmedHIV(+) Serology (n = 22)	Total HIV(+)Serology	Clinical CriteriaOnly (n = 24)
Any CD4+ lymphocyte countCD4+ count <350/µL	48 (28%)30 (18%)	7 (28%)2 (8%)	13 (37%)8 (36%)	20 (33%)10 (21%)	28 (23%)20 (16%)
Any antiretroviral (cARV) therapySpecific cARV regimenRegimen not specified	87 (51%)38 (22%)49 (29%)	12 (48%)3 (12%)9 (36%)	16 (73%)13 (59%)3 (14%)	28 (60%)16 (34%)12 (26%)	59 (48%)22 (18%)*37 (30%)
WHO clinical stage and/or ISSin medical record	171 (100%)	25 (100%)	22 (100%)	47 (100%)	124 (100%)

Of the subsequent confirmed HIV(+) serology 12 were identified at the Comprehensive Care Clinic and 10 at the Department of Radiation Oncology at Kenyatta National Hospital. [Notes: *Statistically significant difference (p = 0.022) between positive serology group (n = 47) and clinical criteria only group (n = 124) with respect to the level strength-of-evidence (Chi-square test)].

Upon referral to two other treatment facilities per NCR policies of the original 146 cases that fulfilled clinical criteria for evidence of HIV infection and or AIDS, an additional 22 confirmed HIV(+) serology cases were identified. This included 12 cases at the Comprehensive Care Clinic and 10 cases at the Department of Radiation Oncology both based at KNH. Data from these two groups of HIV(+) serology cases were reported separately for descriptive purposes, but were combined to compare to those 124 individuals that fulfilled clinical criteria for evidence of HIV infection but did not have a known positive HIV serology. When combined, the HIV(+) serology group [n = 47 (27%)] was not found to be statistically significantly different from the clinical criteria-only group [n = 124 (73%)] for most characteristics, except for rate of those who had a specific cARV regimen [34% in the HIV(+) group compared to 18% in the clinical criteria-only group (see Table 2)] and for gender [51% male in the HIV(+) group compared to 28% male in the clinical criteria-only group (see Table 3)].

**Table 3:** Comparison of demographics and percentage with known HIV-associated cancer cases between those with HIV(+) serology and those who met clinical criteria only.

		HIV Serology			
Patient Characteristics	Total(n = 171)	Initial HIV(+) Serology (n = 25)	Subsequent ConfirmedHIV(+) Serology (n = 22)	Total HIV(+)Serology	Clinical Criteria Only (n = 124)
Male sex	59 (35%)	14 (56%)	10 (45%)	24 (51%)	35 (28%)*
Median age (range) in years	39 (7–82)	41 (7–61)	39 (28–69)	40 (7–69)	39 (8–82)
Histological or cytological basis of diagnosis (otherwise in clinical chart)	142 (83%)	19 (76%)	22 (100%)	41 (87%)	101 (81%)
Known HIV-associated tumor (i.e., KS, cervix, NHL, conjunctiva, and HD)	118 (69%)	17 (68%)	18 (82%)	35 (74%)	83 (67%)

[Notes: *Statistically significant difference (p = 0.005) between positive serology group (n = 47) and clinical criteria only group (n = 124) with respect to the patient characteristic (Chi-square test)].

### Cancer Diagnosis

Primary tumor site(s) were identified per standard registry reporting methodology for all 171 cases (Table 4). Histology reports were verified in 142 (83%), cytology in 6 (3.5%), and the clinical record in the remaining 23 (13.5%) cases respectively. Of the 171 cases, 118 (69%) were tumor types with known association with HIV infection including Kaposi’s sarcoma (n = 55), cervical cancer (n = 42), non-Hodgkin’s lymphoma (n = 15), Hodgkin’s disease (n = 4), and carcinoma of the conjunctiva (n = 2; out of 10 “orbital tumors”). There was a preponderance of female cases 112 (65%) and the median age at cancer diagnosis was 39 years (range 7–82). Two children were registered. An 8-year old boy with Burkitt’s lymphoma meeting HIV clinical criteria; and another 7-year old boy with Kaposi’s sarcoma and with documented HIV(+) serology.

**Table 4:** Tumor types of 171 cancer cases with hierarchal association of HIV infection/AIDS.

		HIV Serology		
Primary Tumor Type	Clinical CriteriaOnly (n = 124)	Initial HIV(+) Serology (n = 25)	Subsequent ConfirmedHIV(+) Serology (n = 22)	Total (n = 171)
***Kaposi’s Sarcoma*** (KS)	37	6	12	55 (32%)
***Cervix***	31	5	6	42 (25%)
***Non-Hodgkin’s lymphoma*** (NHL)	12	3	0	15 (9%)
Head and Neck	8	2	0	10 (6%)
Orbit/***Conjunctiva***	7/2	0	1/0	10 (6%)
Breast	5	4	0	9 (5%)
Stomach	3	1	0	4 (2%)
***Hodgkin’s Disease*** (HD)	1	3	0	4 (2%)
NOS abdomen/carcinoma in situ NOS	3/1	0	0	4 (2%)
Skin (NOS)/Melanoma	2/0	0	0/1	3 (2%)
Uterus/Vulva	1/2	0	0	3 (2%)
Esophagus	2	0	0	2 (1%)
Colon	2	0	0	2 (1%)
Liver	1	0	1	2 (1%)
Kidney/Prostate	0/1	0	1/0	2 (1%)
Brain	0	1	0	1 (<1%)
Leukemia (NOS)	1	0	0	1 (<1%)
Lung	1	0	0	1 (<1%)
Pancreas	1	0	0	1 (<1%)

Of these total cases, 118 (69%) had demonstrable HIV association (i.e., KS, cervix, NHL, conjunctiva and Hodgkin’s disease) and the remaining 53 would be considered non-AIDS-defining cancer. [Notes: ***Bold italics*** – known HIV-associated malignancy; NOS – not otherwise specified].

## Discussion

The Kampala Cancer Registry, which is based in Kyadondo County in Uganda, is among the oldest tumor registries in sub-Saharan Africa and has successfully conducted AIDS-cancer match disease surveillance. [21], [22] In Kenya and for much of sub-Saharan Africa, however, there is limited or no capability for electronic or other linkage of cancer and AIDS cases for disease co-registration. This study to our knowledge represents the *first-of-its-kind*, to pilot a cancer-AIDS match registration strategy based on the merits of clinical strength-of-evidence. Clinical HIV infection data was retrieved in a hierarchal manner upon review of medical records documentation at time of cancer registration. Documentation of positive HIV serology and/or plasma HIV-1 RNA viremia (i.e., viral load) provides unequivocal evidence (*sine qua non*) of HIV infection. Using this as a departure point and uncertain of how frequently these laboratory parameters would be identified in the medical records at time of cancer registration, we chose to develop in a pragmatic and teachable manner, other tiers of evidence for tumor registrars. Drug prescription is frequently, if not always, documented in Kenyan medical records given its reliability since patients have to pay for all their medications or secure medicines through specialty programs and clinics. We chose this as the next tier of evidence. This then afforded the NCR leadership staff and physicians an opportunity to readily train registrars on the different antiretroviral agents and regimens commonly prescribed for HIV infection in Kenya. [23], [24] We next selected other laboratory evidence of HIV infection and in this instance restricted our review to documentation of CD4+ lymphocyte counts. We did not anticipate seeing any documentation of plasma HIV-1 RNA levels and this supposition was indeed confirmed as there were no reports of HIV viral load documented in the medical records. The paucity of laboratory data is also explainable by the recognition that once an HIV diagnosis is established in Nairobi, the bulk of patient care and laboratory monitoring is done in highly specialized clinics. These records are not part of the general medical records. Finally, any historical comment that documented HIV infection by referencing WHO clinical stage or presence of immune suppression syndrome (ISS), which is frequently documented in medical histories, were judged acceptable but was considered the lowest tier of evidence. Knowledge for each of these hierarchal categories is readily taught, comprehended, and implemented for disease registration by cancer registrars.

With this backdrop it is important to recognize the spectrum of malignancy that was identified in this pilot study. The 47 cases with positive HIV serology are indicative of a verifiable 4% [47 HIV(+) cases/1,200 cancer cases)] HIV seroprevalence rate among cancer cases registered and is reflective of the national Kenyan HIV seroprevalence rate, which is on the order of 6.3%. [15], [16] Admittedly, risk of HIV infection is increased in cancer patients given the high incidence of KS, cervical cancer, and NHL in particular in Kenya and throughout sub-Saharan Africa. Thus, the 14% (171 clinical criteria cases/1,200 cancer cases) HIV seroprevalance rate based on strength-of-evidence criteria is likely within an acceptable boundary. Additionally, up to 69% (118) of the 171 total cancer cases identified using our approach have documented associations with HIV infection – KS, cervical cancer, NHL, conjunctival carcinoma, and Hodgkin’s disease. Of the 10 orbital tumors, only two were specifically diagnosed as carcinoma of the conjunctiva, which is associated with HIV infection and immunodeficiency. [25], [26] It is plausible that these 8 other tumors are likely conjunctival in origin, and this would then approximate 74% of cases with clearly established association with HIV infection. Importantly, 87% of cancer cases had verifiable histology or cytology reports that were confirmed in the medical record, which is in keeping with international guidelines for cancer registration.

The remaining 53 (31%) cancer cases (118 tumor types with known HIV association of the 171 total cases) would be considered to be non-AIDS-defining cancer with head and neck (10 cases), breast (9), and orbital tumors (8) being the most frequently encountered primary sites. Additionally, isolated cases of liver, lung, and leukemia were identified and all have been reported in the backdrop of HIV infection. These observations are entirely in keeping with studies originating in the US and Europe since late 1990s to the mid-2000s on the declining incidence of HIV-related tumors, such as KS and NHL in particular, and the increasing burden of non-AIDS-defining cancer. [27]–[34] This is undoubtedly attributed to aging of the HIV-infected population in the developed world and the success of the contemporary cARV therapeutic era in improving immune status and controlling viral replication. Similarly, with roll out of international (e.g., US PEPFAR program) and Kenyan national HIV treatment programs, similar dynamics of an aging HIV-infected patient population and improved cARV coverage in Kenya and the rest of the African continent, the emergence of non-AIDS-defining cancers is likely just on the horizon. [5] In developed portions of the world, strategies are already being implemented for screening HIV-infected patients at increased risk for non-AIDS-defining malignancy [35].

There are important limitations of our study. Among them is the very small sample size derived over a 3-month period of time; review of medical records that despite our hierarchal strength-of-evidence approach had limited documentation of HIV-serology; other laboratory tests indicative of HIV infection were scant, which precluded any correlation of immunodeficiency (level of CD4+ count) with type of cancer diagnosis; and no documentation of plasma HIV-1 RNA levels. For the most part the 47 cases that were confirmed HIV(+) versus the clinical criteria only group (n = 124) were fairly comparable. The only statistically significant difference in the characteristics we analyzed were predominance of male sex and precise documentation of cARV regimen in the HIV(+) versus the clinical-criteria only groups. These differences are not clinically significant and can be readily explained on the basis that up to 25% of the HIV(+) group, the largest proportion, were derived from the records of the KNH Comprehensive Care Clinic. This clinic is a comprehensive HIV/AIDS treatment center in Nairobi.

In summary, this pilot demonstration of a hierarchal, clinical strength-of-evidence approach for cancer-AIDS registration in Kenya establishes feasibility, is readily adaptable, pragmatic, and does not require additional resources for critically under-staffed cancer registries. Cancer is an emerging public health challenge in Africa, and African nations need to develop well designed population-based studies in order to better define the impact and spectrum of malignant disease in the backdrop of HIV infection and AIDS. [36], [37] As we approach the midpoint of the fourth decade of the AIDS pandemic, the emergence of non-AIDS-defining cancer in HIV-infected individuals, nearly a third of cases based on this survey in Nairobi, is likely to evolve in Africa as it did around the turn of the century in the developed world.
